# Beyond the Surgery: The Impact of Coping Strategies on Persistent Pain After Rotator Cuff Repair

**DOI:** 10.3390/jcm13216584

**Published:** 2024-11-01

**Authors:** Daniela Brune, David Endell, Steven Z. George, Robert Edwards, Markus Scheibel, Asimina Lazaridou

**Affiliations:** 1 Research and Development—Shoulder and Elbow Surgery, Schulthess Clinic, 8008 Zürich, Switzerland; daniela.brune@kws.ch; 2 Department of Shoulder and Elbow Surgery, Schulthess Clinic, 8008 Zürich, Switzerland; david.endell@kws.ch; 3 Department of Orthopedic Surgery, Duke Clinical Research Institute, Duke School of Medicine, Durham, NC 27710, USA; steven.george@duke.edu; 4 Department of Anesthesiology, Brigham & Women’s Hospital and Harvard Medical School, Boston, MA 02115, USA; rredwards@bwh.harvard.edu; 5 Center for Musculoskeletal Surgery, Charité-Universitätsmedizin, 10117 Berlin, Germany

**Keywords:** rotator cuff, shoulder, coping skills, pain, postoperative, mediation analysis

## Abstract

**Background:** Rotator cuff repair is widely recognized as one of the most painful orthopedic surgeries, yet postoperative pain management in these patients is often underexplored. This study aimed to explore the relationship between pain outcomes and functional recovery six months after arthroscopic rotator cuff repair (ARCR), with a focus on the role of different pain coping mechanisms as mediators. **Methods:** This study included 83 patients that underwent rotator cuff repair. Pain levels were assessed using the Brief Pain Inventory (BPI-SF), while shoulder function was evaluated using the Oxford Shoulder Score (OSS). Coping strategies, including self-statements, ignoring pain, distraction, and praying, were examined in relation to pain severity and interference and were assessed with the Coping Strategies Questionnaire-Revised (CSQ-R). Simple and parallel mediation analyses were performed using the PROCESS macro to assess the mediating effects of coping mechanisms on the relationship between pain intensity, pain interference, and postoperative OSS. **Results:** Post-surgery, patients showed a significant improvement in OSS (from 29 ± 9 to 42 ± 6). At six months, 24% of patients reported chronic postsurgical pain (CPSP), defined as a pain severity score of 3 or higher. Correlation analyses revealed that OSS was negatively associated with pain catastrophizing (r = −0.35, *p* < 0.01) and praying (r = −0.28, *p* < 0.01). OSS was significantly negatively associated with pain severity (r = −0.54; *p* < 0.01) and pain interference (r = −0.51, *p* < 0.01). Mediation analysis demonstrated that coping self-statements significantly mediated the relationship between pain interference and shoulder function (a*b = 0.5266 (BootSE = 0.2691, 95% CI [0.1010, 1.1470]), emphasizing the important role of cognitive strategies in supporting recovery outcomes. **Conclusion:** Patients engaging in adaptive coping strategies, particularly coping self-statements, reported better functional outcomes. The findings underscore the importance of targeted interventions focusing on effective pain coping mechanisms to improve recovery post-ARCR.

## 1. Introduction

Rotator cuff tears are among the most common shoulder injuries, leading to functional impairment, pain, and severe limitations in daily activities. Pain is the primary manifestation of a rotator cuff tear, driving patients to seek surgical intervention with the expectation of relief following repair [1]. The incidence of rotator cuff repairs has risen considerably, with approximately 58 procedures per 100,000 people performed annually in the United States by 2006 [2], 131 per 100,000 in Finland by 2011 [3], and 62 per 100,000 in Italy by 2014 [4]. Arthroscopic rotator cuff repair (ARCR) is considered the gold standard for treating these injuries and has consistently demonstrated effectiveness in reducing postoperative pain and improving both functional and subjective patient outcomes [5,6]. However, ARCR is often associated with a higher degree of acute postoperative pain, especially in the initial weeks following surgery [7]. This pain is typically most intense during the first six weeks [8,9] and gradually diminishes thereafter [10]. Effective management of this postoperative pain is crucial, and guidelines have been established to provide clinicians with evidence-based strategies to enhance short-term pain relief following rotator cuff repair [11]. Improvements in postoperative functional scores are often indicative of effective pain management and recovery [12,13], allowing surgeons to adjust pain management strategies if patients’ functional outcomes or pain levels remain elevated after surgery [14].

Pain that persists for more than 3 months after surgery is considered chronic postsurgical pain (CPSP) [15] and presents a significant challenge in postoperative care. There is no clear transition period between postoperative pain and CPSP, and the underlying pathophysiology in the transition from acute to chronic pain is complex and multifactorial [16]. In Europe, the incidence of moderate to severe CPSP one year after rotator cuff surgery is approximately 13.5% [17]. This rate is consistent with the incidence of CPSP observed in the broader surgical population [18]. One year after ARCR, higher pain scores are more commonly linked to psychosocial factors rather than specific structural details of the tear [19]. These psychosocial predictors include preoperative pain levels, narcotic use, and emotional well-being. Poor mental health, including high levels of depression, anxiety, and pain catastrophizing, is associated with worse outcomes and elevated pain levels following shoulder surgery [20,21]. Specifically, pain catastrophizing has been consistently linked to higher pain intensity and prolonged recovery, highlighting the importance of psychological assessment [22]. Conversely, optimism has been shown to enhance pain tolerance and lead to better outcomes for shoulder pain [23].

Many pain models underscore the role of coping responses in adjusting to chronic conditions, with evidence suggesting that adaptive strategies, such as acceptance, reduce disability and depression [24,25]. Coping strategies are categorized into frameworks such as problem- versus emotion-focused, cognitive versus behavioral, and active versus passive, each addressing distinct aspects of self-regulation in response to pain [26]. Recent research suggests that active coping strategies, including positive self-statements and seeking social support, are generally associated with improved outcomes, whereas passive approaches, such as avoidance and catastrophizing, tend to exacerbate pain severity and impair functioning [26,27].

While research has extensively explored how protective factors influence outcomes in various orthopedic surgeries, such as knee and hip replacements, similar studies focusing on shoulder surgeries are less common. For instance, after arthroscopic knee surgery, problem-focused strategies and positive self-statements have been found to enhance pain tolerance and improve surgical outcomes [28,29]. Additionally, dysfunctional coping and pain catastrophizing are linked to a higher risk of chronic pain in hip and knee replacements [30]. Poor emotional health and inadequate coping skills also correlate with suboptimal functional outcomes across multiple orthopedic procedures, including spine surgeries [31].

Given these complexities, there is a call to investigate how pain coping mechanisms affect outcomes after ARCR, especially concerning CPSP. Effective pain coping strategies are vital for managing both pain and psychological distress, particularly in individuals with chronic pain. Strengthening cognitive and behavioral coping through multidisciplinary approaches is essential for successfully treating chronic primary pain, leading to improved functional and mental health outcomes [32]. Therefore, this study primarily aimed to examine how pain outcomes and functional recovery relate to each other six months after ARCR, with an emphasis on the role of different pain coping mechanisms as mediators.

## 2. Materials and Methods

### 2.1. Participants and Procedure

This was a cross-sectional study on a subset of patients from a local single-center Rotator Cuff Registry based at an orthopedic private clinic (BASEC No. 2014-0253). Eligibility criteria for this study were as follows: participants must have undergone either partial or total arthroscopic rotator cuff surgery between October 2023 and March 2024 and must have consented to the use of their health-related data for research purposes. Exclusion criteria included individuals with language barriers, those who had revision surgery, or those with incomplete preoperative or postoperative questionnaire data. Eligible participants were invited to complete a single survey six months after their ARCR. This survey focused on coping mechanisms during the recovery period and pain assessment using patient-reported outcome measurements (PROMs). The six-month follow-up was selected as it represents a critical timeframe in orthopedic research for evaluating the persistence of pain and assessing the durability of intervention effects [33,34]. Additional information, including baseline characteristics, surgical details, and postoperative Oxford Shoulder Score (OSS) scores, was collected from the local Rotator Cuff Registry. A total of 165 patients were screened and invited to participate in the study.

ARCRs were conducted following standardized, clinic-specific, and international guidelines with patients positioned in beach chair position under general anesthesia. A standard 3-phase postoperative physical therapy regimen was prescribed and included immobilization on an abduction pillow with passive mobilization for the first 6 weeks, active mobilization combined with coordination training for the next 4 weeks, and progressive resistance exercises.

All participants enrolled in this study provided their written informed consent for inclusion in both the Rotator Cuff Registry and the study-specific questionnaire.

### 2.2. Measures

#### 2.2.1. Sociodemographic and Clinical Data

Sociodemographic information included age at the time of surgery, sex, marital status, education level, body mass index (BMI), and the American Society of Anesthesiologists (ASA) classification. Negative effects were measured using the “Anxiety/Depression” dimension of the EQ-5D-5L scale [35], which ranges from level 1 (“I am not anxious or depressed”) to level 5 (“I am extremely anxious or depressed”). Clinical data included rotator cuff tear severity, tear pattern, and additional procedures, including interventions on the long head of the biceps tendon and acromioplasty. All data were extracted from the local Rotator Cuff Registry.

#### 2.2.2. Oxford Shoulder Score (OSS)

The OSS is a 12-item, unidimensional patient-reported outcome measurement (PROM) specifically designed to assess outcomes following shoulder surgery [36]. The items cover various aspects of shoulder pain and the impact of shoulder function on daily activities. In the original version of the OSS, patients rate each item on a scale from 1 (best/fewest symptom) to 5 (worst/most severe). The scores are then summed to produce the total OSS ranging from 12 (best, indicating no pain or functional impairment) to 60 (worst outcome), with the higher scores reflecting a greater degree of disability [36].

In 2009, the OSS scoring method was revised so that each of the 12 items are now scored from 4 (best/fewest symptoms) to 0 (worst/most severe). This adjustment results in a total score ranging from 0 to 48, where lower scores indicate a higher degree of disability [36]. We utilized this revised scoring method for its simpler interpretation. The German version of the OSS, which was adapted and validated for clinical use, was translated according to internationally standardized guidelines [12].

#### 2.2.3. German Version of the Brief Pain Inventory Short Form (BPI-SF)

BPI-SF [37] is a widely recognized and comprehensive tool for pain assessment, established as a standard use in multinational studies [38]. This short, self-administered questionnaire is user-friendly and effective in the evaluation of both pain intensity and pain-related functional impairment. The German version of the BPI has demonstrated comparable validity and reliability to the original [37].

The BPI is a 15-item measure that consists of two multi-item sub-scales that measure pain intensity and pain interference [39,40]. Pain intensity is measured through four items using a Numeric Rating Scale (NRS) ranging from 0 (“no pain”) to 10 (“pain as bad as you can imagine”). Patients are asked to rate their pain across four scenarios: (1) worst in the last 24 h, (2) least in the last 24 h, (3) average pain, and (4) current pain.

Pain interference evaluates the extent to which pain interferes with the patient’s daily functioning. This is also assessed using the NRS, where patients rate the interference on a scale from 0 (“does not interfere”) to 10 (“completely interferes”) across seven functional areas (mood, walking ability, normal work (including housework), relations with other people, sleep, and enjoyment of life).

#### 2.2.4. Pain Coping Strategies Questionnaire-Revised (CSQ-R)

The CSQ is one of the most widely used tools internationally for assessing pain coping strategies. It is particularly effective in evaluating how patients with chronic musculoskeletal pain manage their pain through various cognitive and behavioral techniques [41]. The German version of the CSQ-R was validated and shown to be a feasible and reliable outcome measure for this population [41].

The CSQ-R is a 27-item self-report questionnaire designed to assesses six key cognitive behaviors: distraction, catastrophizing, ignoring pain sensations, distancing from pain, and praying [42,43]. Patients are asked to rate the frequency with which they use each of these coping strategies on a 6-point Likert scale, ranging from 0 (“Never do this”) to 6 (“Always do this”).

### 2.3. Power Analysis

A power analysis was conducted using G*Power software (version 3.1.9.6, developed by the University of Düsseldorf, Germany) [44,45] to determine the appropriate sample size required to achieve adequate power for detecting statistically significant effects. The analysis indicated that a minimum sample size of n = 55 patients is necessary to ensure 80% power for detecting a statistically significant relationship (*p* < 0.05) in a model that includes one independent variable (either pain severity or pain interference), six mediation variables (CSQ-R coping mechanisms), and two covariates (baseline OSS and preoperative negative effect). This sample size allows for the robust testing of the hypothesized mediation relationships within this study.

### 2.4. Statistical Analysis

Descriptive statistics were presented as means and standard deviations (SDs) for continuous variables and as percentages for categorical variables. Preliminary analyses included Pearson’s correlation analysis to identify associations among coping strategies, pain outcomes (pain severity and pain interference), and the OSS postoperatively.

To predict OSSs, multivariate linear regressions were used in this exploratory analysis to examine the relationships between OSS, pain intensity, pain interference, and coping strategies. Additionally, to assess the mediating effects of coping mechanisms, in the relationship between pain intensity and interference and postoperative OSS, both simple and parallel mediation analyses were conducted using the PROCESS macro (version 4.3.1, written by Andrew F. Hayes, available from www.processmacro.org (accessed on 1 August 2024)). This macro employs ordinary least squares (OLSs) regression to calculate mediation effects and uses bootstrapping to generate confidence intervals (CIs). Bias-corrected bootstrap CIs were based on 10,000 bootstrap samples with a 95% confidence level. A mediating effect was considered significant if the CI did not include zero. Bootstrapping is especially valuable because it does not rely on assumptions about the shape of the variable distributions or the sampling distribution of the statistics [46].

## 3. Results

### 3.1. Sample Characteristics

A total of 165 patients were screened and invited to participate in this study. A total of 83 patients completed the questionnaire, and the final sample size consisted of 32 females (39%) and 51 males (61%), with a mean age of 61 ± 10 years at the time of surgery. All patients underwent arthroscopic rotator cuff repair, with an average surgery duration of 88 ± 34 min. Patients were treated for partial tears (19%), single full-thickness tears (31%), involvement of two or three tendons with only one fully ruptured (22%), and massive tears (28%). The most common tear pattern was isolated Supraspinatus (43%), followed by combined Supraspinatus and Subscapularis (23%). Additional procedures included Long Head Biceps Tendon Tenodesis in 67% of cases, while acromioplasty was performed in 86% of patients (Table 1). The baseline OSS was 29 ± 9 (on a scale of 48 to 0, with lower scores indicating a higher degree of disability), while the postoperative OSS improved to 42 ± 6. Baseline pain, as measured by NRS, averaged 5.8 ± 2.5, decreasing to 1.7 ± 1.8 postoperatively.

Six months after surgery, patients reported a mean pain severity of 1.5 ± 1.8 and a pain interference score of 1.3 ± 1.9. A total of 20 patients (24%) reported a pain severity score of 3 or higher, indicating the presence of CPSP. The most frequently employed coping strategies were ignoring pain (mean ± SD: 2.8 ± 1.6), coping self-statements (2.7 ± 1.6), and distraction (2.5 ± 1.9). In contrast, the least utilized strategies were praying (0.9 ± 1.6) and catastrophizing (1.0 ± 1.1) (Table 2).

### 3.2. Correlation

The Pearson correlations (Table 2) demonstrated that a negative effect was negatively correlated with OSS (r = −0.53, *p* < 0.01), indicating that a higher negative effect is associated with worse shoulder function. A negative effect is also positively correlated with pain severity (r = 0.29, *p* < 0.01) and pain interference (r = 0.33, *p* < 0.01), suggesting that individuals with a larger negative effect tend to experience more severe and disruptive pain.

OSS was significantly negatively associated with pain severity (r = −0.54; *p* < 0.01) and pain interference (r = −0.51, *p* < 0.01). This indicates that worse shoulder function is associated with higher pain severity and greater interference in daily activities. Among the six coping mechanisms examined, catastrophizing stands out as the one that is significantly negatively correlated with OSS (r = −0.35, *p* < 0.01), representing an association between higher levels of catastrophizing and poorer shoulder function. Furthermore, pain catastrophizing is strongly positively correlated with both pain severity (r = 0.55, *p* < 0.01) and pain interference (r = 0.52, *p* < 0.01), suggesting that individuals who engage in more catastrophizing tend to experience more intense pain and greater interference in their daily lives. Praying was negatively correlated with OSS (r = −0.28, *p* < 0.01), indicating that individuals who use praying as a coping mechanism demonstrate poorer shoulder function. Additionally, those who have greater pain interference tend to engage more in praying activities (r = 0.24, *p* < 0.05).

### 3.3. Mediation Analysis

The parallel mediation analysis for pain severity examined six potential mediators: catastrophizing, coping self-statements, distancing from pain, distraction, ignoring pain, and praying, while controlling for baseline OSSs and negative effect. The total effect of pain severity on OSS was significant (c = −1.4, *p* < 0.001, 95% CI [−2.0043, −0.7963]), indicating that higher pain severity is associated with reduced shoulder function. The direct effect of pain severity on OSS, after accounting for the mediators, remained significant (c’ = −1.3, *p* = 0.0002, 95% CI [−2.0177, −0.6670]).

The total indirect effect of pain severity on OSS through the combined mediators was not statistically significant (a*b = −0.0554, BootSE = 0.2308, 95% CI [−0.5092, 0.3925]), suggesting that these mediators do not significantly explain the relationship between pain severity and shoulder function.

**Figure 1:**
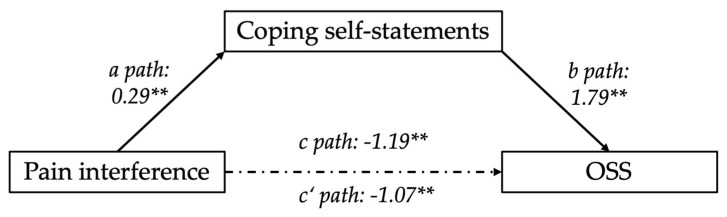
Mediation diagram; coping self-statements mediating the relationship between pain interference and OSS controlling for baseline negative effect and OSS. Path a, b, c, and c’ are path coefficients representing unstandardized regression weights. The c path coefficient represents the total effect of pain interference on OSS. The c’ path coefficient refers to the direct effect of pain interference on OSS. ** indicates *p* < 0.01.

A subsequent parallel mediation model for pain interference, exploring the same six potential mediators (catastrophizing, coping self-statements, distancing from pain, distraction, ignoring pain, and praying) and controlling for baseline OSSs and negative effect, accounted for 35% of the variance in OSS (R^2^ = 0.3521). Among the mediators, coping self-statements emerged as a significant mediator. The parallel mediation analysis revealed that the indirect effect of coping self-statements was significant a*b = 0.5266 (BootSE = 0.2691, 95% CI [0.1010, 1.1470]) (Figure 1). This result demonstrates that coping self-statements mediate the relationship between pain interference and shoulder function, where higher levels of coping self-statements are associated with better shoulder function, improving OSS.

In contrast, the other mediators (catastrophizing, distancing from pain, distraction, ignoring pain, and praying) did not significantly mediate this relationship, as their respective bootstrap confidence intervals included zero. The total effect (c = −1.1907, *p* = 0.0003, 95% CI [−1.8203, −0.5612]) of pain interference on OSS was significant, suggesting that higher pain interference aligns with reduced shoulder function. The direct effect of pain interference on OSS, after accounting for these mediators, was statistically significant (c’ = −1.0671, *p* = 0.0034, 95% CI [−1.7690, −0.3653]). This suggests that cognitive strategies may play a central role in how pain interference affects shoulder function.

## 4. Discussion

The aim of this study was to assess the relationship between clinical pain and shoulder function six months post-ARCR. Specifically, we assessed the mediating role of modifiable cognitive and behavioral strategies, including distraction, catastrophizing, ignoring pain sensations, distancing from pain, and praying. Our main findings highlight a strong link between pain severity, interference, and shoulder function. Notably, coping self-statements were identified as a mediator between the relationship of pain interference and shoulder function. This suggests that the influence of pain interference on shoulder function may be channeled through cognitive strategies, particularly coping self-statements, rather than through the other mediators assessed in this model. This might imply that although pain interference negatively affects overall function, patients who employ positive coping self-statements can mitigate some of the negative impact of pain on their function.

Prior research in orthopedics has underscored the significance of coping mechanisms in postsurgical outcomes [47]. For example, enhancing adaptive coping strategies during the early recovery phase can improve postsurgical outcomes such as patient’s quality of life [48] and pain [23]. Conversely, maladaptive coping such as catastrophizing has been linked to poorer outcomes, including increased pain perception and recued function [49]. Coping mechanisms, which include both cognitive and behavioral strategies, are essential tools individuals use to manage the emotional and physical stress associated with pain [27,28,32,50,51,52,53,54,55]. Cognitive strategies involve mental techniques aimed at altering the perception of pain, such as distraction or positive self-talk, whereas behavioral strategies encompass actions like physical activities or relaxation to manage pain [56]. Distinguishing between adaptive and maladaptive coping mechanisms is crucial, as adaptive strategies generally lead to better pain management, psychological well-being, and functional outcomes, while maladaptive strategies often exacerbate pain perception and disability [50,54,57].

In our cohort, the most frequently utilized coping strategies by our patient cohort were coping self-statements, ignoring pain, and distraction. This aligns with previous research indicating that patients often turn to strategies that provide immediate psychological relief [58,59]. Despite ignoring pain typically being classified as maladaptive [60,61], its common use in our study suggests that patients may still perceive some short-term benefit. For example, avoiding pain might stem from not wanting to complain, fearing side effects, or being reluctant to take medication [61]. However, the reliance on adaptive coping mechanisms such as coping self-statements and distraction is encouraging, as these strategies are known to enhance psychological resilience and reduce stress perception [52,55,62,63]. This finding aligns with recent studies emphasizing the importance of adaptive strategies in mitigating the negative effects of maladaptive coping mechanisms. For instance, optimism has been shown to reduce the negative impact of pain catastrophizing on shoulder function, even if it does not directly influence pain intensity [23]. This might suggest that fostering positive psychological constructs, such as optimism and self-efficacy, may be beneficial for patients recovering from surgery.

Pain catastrophizing, a well-studied cognitive mechanism in pain research, involves an exaggerated negative focus on pain, often leading to increased pain intensity, emotional distress, and disability [53]. Previous studies have consistently shown that catastrophizing predicts higher levels of pain and disability in chronic pain patients [49,64,65]. Also, in a longitudinal analysis, higher levels of pain catastrophizing at baseline predicted greater pain intensity and disability over time [66,67] in patients with chronic shoulder pain. Surprisingly, in our study, catastrophizing did not emerge as a significant mediator in the relationship between pain severity and shoulder function. This may be due to its relatively low prevalence as a coping mechanism in our cohort, potentially reducing its impact as a mediator. While much research in orthopedic surgery has focused on the negative aspects of pain, such as catastrophizing, there has been less emphasis on positive coping strategies. Specifically, the role of adaptive coping mechanisms, such as coping self-statements, has been underexplored in the context of rotator cuff surgeries, highlighting a gap in the current literature.

Moreover, there is growing recognition that a holistic approach to pain management, incorporating broader concepts such as mindfulness, acceptance, and psychological flexibility, may be more effective than focusing solely on reducing catastrophizing [68,69]. Mindfulness and acceptance-based interventions encourage patients to engage with their pain in a non-judgmental and accepting manner, potentially reducing psychological distress and improving overall quality of life. These interventions align with the adaptive strategies observed in our study, particularly the use of coping self-statements, which may represent an initial step toward cultivating a more mindful and accepting approach to pain management. In a study evaluating the effectiveness of psycho-educational interventions in pain management after shoulder surgery using a pain management booklet and coaching, those interventions help patients to manage their pain effectively [70].

When interpreting the results of this study, several limitations must be considered when interpreting the results. The Oxford Shoulder Score (OSS) is a valuable tool for assessing shoulder pain and daily function; however, as a patient-reported outcome measure (PROM), it may be susceptible to recall bias, which could affect the accuracy of symptom reporting. The Coping Strategies Questionnaire-Revised (CSQ-R), used to evaluate coping mechanisms, has a complex factor structure and has shown inconsistencies across studies. This variability suggests that the CSQ-R may not consistently capture coping strategies across different clinical populations [41,42,43,56]. Furthermore, its length could burden patients, affecting response accuracy. Another important limitation is the cross-sectional nature of this study, which does not allow for establishing cause-and-effect relationships but rather highlights associations between pain, function, and coping mechanisms. Multidimensional measurements, like physical performance tests or quantitative sensory testing, are recommended for more consistent and accurate and comprehensive assessments of pain and function [71]. While our study’s sample size of 83 patients was sufficient for detecting significant associations, it may not fully represent the diversity of the broader population, potentially limiting the generalizability of our findings. Despite these limitations, this study offers valuable insights, but its conclusions should be interpreted with caution considering these potential sources of bias.

## 5. Conclusions

In conclusion, our study underscores the critical role of adaptive cognitive strategies, particularly coping self-statements, in mitigating the impact of pain interference on shoulder function in patients recovering from ARCR. While maladaptive strategies like catastrophizing are well documented, our findings suggest that fostering adaptive coping mechanisms could be just as, if not more, vital for improving recovery outcomes. Collectively, there is a call for integrating these adaptive strategies into pain management approaches to better address the broader psychological context of pain and enhance overall recovery.

## Figures and Tables

**Table 1:** Baseline patient characteristics (n = 83).

Age, mean ± SD		61 ± 10
Female sex, n (%)		32 (39%)
Marital status, n (%)		
	Single	7 (8%)
	Partnership/married	63 (76%)
	Divorced	8 (10%)
	Widowed	5 (6%)
Education level, n (%)		
	Compulsory education	6 (7%)
	Secondary level education	31 (37%)
	Tertiary level education	46 (55%)
BMI, Mean ± SD		26 ± 4
ASA classification, n (%)		
	ASA I	15 (18%)
	ASA II	57 (69%)
	ASA III	11 (13%)
Negative effect (score range: 1–5), mean ± SD		1.4 ± 0.8
OSS (score range: 0–48), mean ± SD		29 ± 9
Baseline NRS pain (0–10), mean ± SD		5.8 ± 2.5
Rotator cuff tear severity, n (%)		
	Partial tear	16 (19%)
	Single full tear	26 (31%)
	Two or three tendons (only one full)	18 (22%)
	Massive tear	23 (28%)
Tear pattern, n (%)		
	ISP	1 (1%)
	SSC	3 (4%)
	SSP	36 (43%)
	SSP and ISP	10 (12%)
	SSP and SSC	19 (23%)
	SSP and ISP and SSC	14 (17%)
Long head biceps tendon procedure, n (%)		
	No intervention	16 (19%)
	Tenotomy	22 (13%)
	Tenodesis	56 (67%)
Acromioplasty performed, n (%)		71 (86%)

Note. Descriptive statistics are presented as means and standard deviations (SDs) for continuous variables and as percentages for categorical variables.

**Table 2:** Pearson’s correlation analysis results at 6 months follow-up.

	Mean ± SD	1	2	3	4	5	6	7	8	9	10
1. Negative effect (1–5)	1.2 ± 0.6	1.00									
2. OSS (0–48)	42 ± 6	−0.53 **	1.00								
3. Distraction (0–6)	2.5 ± 1.9	0.03	−0.08	1.00							
4. Catastrophizing (0–6)	1.0 ± 1.1	0.36 **	−0.35 **	0.26 *	1.00						
5. Ignoring pain (0–6)	2.8 ± 1.6	−0.02	0.00	0.49 **	0.10	1.00					
6. Distancing pain (0–6)	1.3 ± 1.6	0.00	−0.11	0.69 **	0.17	0.46 **	1.00				
7. Coping self-statements (0–6)	2.7 ± 1.6	0.00	0.02	0.60 **	0.38 **	0.74 **	0.58 **	1.00			
8. Praying (0–6)	0.9 ± 1.6	0.21	−0.28 **	0.18	0.18	0.03	0.11	0.10	1.00		
9. Pain severity (0–10)	1.5 ± 1.8	0.29 **	−0.54 **	0.11	0.55 **	0.02	0.03	0.18	0.07	1.00	
10. Pain interference (0–10)	1.3 ± 1.9	0.33 **	−0.51 **	0.20	0.52 **	0.09	0.14	0.30 **	0.24 *	0.72 **	1.00

Note. Mean and SD are used to represent mean and standard deviation, respectively. * indicates *p* < 0.05; ** indicates *p* < 0.01.

## Data Availability

The datasets generated and analyzed during the current study are not publicly available due to privacy and ethical restrictions but are available from the corresponding author on reasonable request. All data will be shared in compliance with institutional guidelines and data-sharing agreements.
